# Opportunities for increased reproducibility and replicability of developmental neuroimaging

**DOI:** 10.1016/j.dcn.2020.100902

**Published:** 2020-12-17

**Authors:** Eduard T. Klapwijk, Wouter van den Bos, Christian K. Tamnes, Nora M. Raschle, Kathryn L. Mills

**Affiliations:** a Erasmus School of Social and Behavioral Sciences, Erasmus University Rotterdam, the Netherlands; b Institute of Psychology, Leiden University, Leiden, the Netherlands; c Leiden Institute for Brain and Cognition, Leiden, the Netherlands; d Department of Psychology, University of Amsterdam, Amsterdam, the Netherlands; e Max Planck Institute for Human Development, Center for Adaptive Rationality, Berlin, Germany; f PROMENTA Research Center, Department of Psychology, University of Oslo, Norway; g NORMENT, Division of Mental Health and Addiction, Oslo University Hospital & Institute of Clinical Medicine, University of Oslo, Norway; h Department of Psychiatry, Diakonhjemmet Hospital, Oslo, Norway; i Jacobs Center for Productive Youth Development at the University of Zurich, Zurich, Switzerland; j Department of Psychology, University of Oregon, Eugene, OR, USA

**Keywords:** Development, Open science, Sample size, Cognitive neuroscience, Transparency, Preregistration

## Abstract

Many workflows and tools that aim to increase the reproducibility and replicability of research findings have been suggested. In this review, we discuss the opportunities that these efforts offer for the field of developmental cognitive neuroscience, in particular developmental neuroimaging. We focus on issues broadly related to statistical power and to flexibility and transparency in data analyses. Critical considerations relating to statistical power include challenges in recruitment and testing of young populations, how to increase the value of studies with small samples, and the opportunities and challenges related to working with large-scale datasets. Developmental studies involve challenges such as choices about age groupings, lifespan modelling, analyses of longitudinal changes, and data that can be processed and analyzed in a multitude of ways. Flexibility in data acquisition, analyses and description may thereby greatly impact results. We discuss methods for improving transparency in developmental neuroimaging, and how preregistration can improve methodological rigor. While outlining challenges and issues that may arise before, during, and after data collection, solutions and resources are highlighted aiding to overcome some of these. Since the number of useful tools and techniques is ever-growing, we highlight the fact that many practices can be implemented stepwise.

## 1 Introduction

In recent years, much has been written about reproducibility and replicability of results being lower than desired in many fields of science (Ioannidis, 2005; Munafò et al., 2017), including in cognitive neuroscience (Poldrack et al., 2017). Reproducibility refers to the ability to obtain the same results using the same data and code, while replicability is the ability to obtain consistent results using new data (Barba, 2018; Nichols et al., 2017). What will count as consistent results and thus form a successful replication is up for debate (Cova et al., 2018; Maxwell et al., 2015; Open Science Collaboration, 2015; Zwaan et al., 2018). For example, one might come to different conclusions about replicability when using statistical significance (e.g., *p* < .05) as a criterion, when comparing the effect sizes of the original and replication study, or when meta-analytically combining effect sizes from the original and replication study (Open Science Collaboration, 2015). In the context of neuroimaging, another complication is the use of qualitatively defined brain regions that may vary from study to study, making it hard to establish whether an effect has been replicated (Hong et al., 2019). Similarly, a distinction is often made between direct replications, in which all major features of the original study are recreated, and conceptual replications, in which changes are made to the original procedure to evaluate the robustness of a theoretical claim to such changes (Zwaan et al., 2018). When we refer to replicability throughout this paper, we use the term in a broad sense of any attempt to establish the consistency of developmental cognitive neuroscience effects using new data.

It has been suggested that low statistical power, undisclosed flexibility in data analyses, hypothesizing after the results are known, and publication bias, all contribute to the low rates of reproducibility and replicability (Bishop, 2019; Munafò et al., 2017). The field of developmental neuroimaging is not immune to the issues that undermine the reproducibility and replicability of research findings. In fact, there are several issues that may be even more pronounced in, or specific to, developmental neuroimaging. For example, recruiting sufficiently large sample sizes is challenging because of the vulnerability of younger populations, and the associated challenges in recruitment and testing. On top of that, to disentangle individual variation from developmental variation, higher numbers of participants are needed to represent different age ranges. If we expect an age effect for a specific psychological construct, the sample size has to be sufficient per age category and not simply the power across the whole sample as would be assumed in an adult group. Examples that are specific for neuroimaging studies include the widely observed problem of greater in-scanner motion with younger age that could confound results, including observed developmental patterns (Blumenthal et al., 2002; Satterthwaite et al., 2012; Ducharme et al., 2016). Moreover, neuroimaging studies typically involve large numbers of variables and a multitude of possible choices during data analyses, including image quality control, the choice of specific preprocessing parameters and statistical designs. A failure to describe these choices and procedures in sufficient detail can vastly reduce the likelihood of obtaining reproducible and replicable results.

**Fig. 1:**
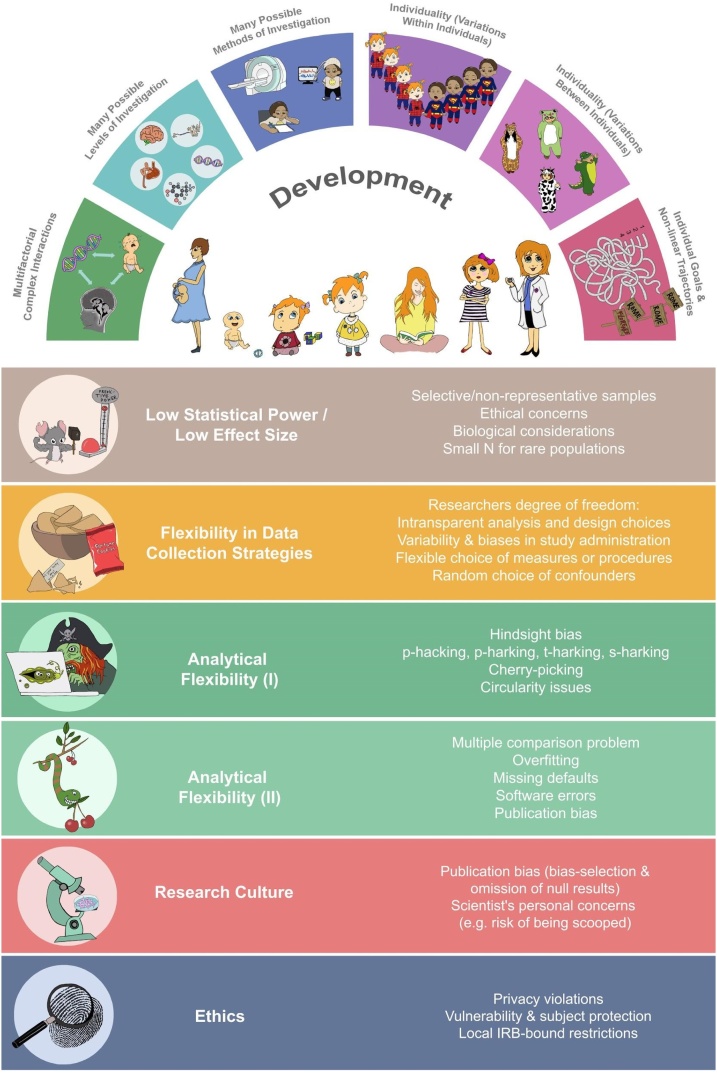
Graphical overview of challenges in the field of developmental cognitive neuroscience. The upper panels represent how development itself is a result of many complex, interacting processes, that it may be described on different levels and studied using different methodologies. Studying development also requires assessment of individuals over time, considering individual variations within and between individuals over time. The lower rectangular boxes depict a summary of challenges to reproducibility and replicability for developmental cognitive neuroscience studies more generally (Illustrations by N.M. Raschle).

In the current review, we outline a number of issues threatening reproducibility and replicability of findings in developmental neuroimaging. Our ultimate goal is to foster work that is not only reproducible and replicable but also more robust, generalizable, and meaningful. At some points, we will therefore also discuss ways to improve our science that might not be directly related to reproducibility and replicability. We will consider issues broadly related to statistical power and flexibility and transparency in data analyses. Given our background, we will focus mainly on examples from structural and functional neuroimaging. Although we do not want to equate cognitive neuroscience with MRI-based measurements, we believe much can be generalized to other modalities used in the broader field of developmental cognitive neuroscience. Fig. 1 summarizes challenges that are specific to the study of development and those that are affecting reproducibility and replicability more broadly. These topics will be picked up later on in Table 1 in more depth. We discuss issues that may arise before, during and after data collection and point to potential solutions and resources to help overcome some of these issues. Importantly, we consider solutions that can be implemented stepwise and by researchers with limited resources such as those early in their career.

**Table 1:** Selective overview of challenges in the field of developmental cognitive neuroscience.

Phase of study		Practical, technical and ethical issues hindering reproducibility & replicability					Potential or previously suggested solutions	Useful links/selected examples	
	**STATISTICAL POWER**								
**1. To consider prior to & throughout data collection**				**Low statistical power / low effect size**		**Power analysis**		G*Power; NeuroPowerTools; BrainPower; fmripower	
						If *no prior reliable data* exists, consider a “smallest effect size of interest’’ consistent with the broader psychological community (e.g., ∼.10 - .30; according to Gignac and Szodorai, 2016)			
						Use of age-adequate and appealing **protocols** to increase power			
						**Sequential interim analyses** (e.g., transparent data peeking to determine cut-off point; Lakens, 2014)			
	**Selective, small or non-representative samples**								
				**Selective/non-representative samples** (e.g., Western, educated, industrialized, rich and democratic (WEIRD) population)		**Measurement invariance tests** (e.g., Fischer and Karl, 2019)			
						**Diversity considerations** in study design & interpretation			
				**Small N due to rare population** (e.g., patients or other populations more challenging to recruit)		Strong ***a priori* hypothesis** (e.g., adjust search space on *a priori*-defined ROIs; caution: (s)harking)			
						Increase **power within subjects** (e.g., consider fewer tasks with longer duration)			
						Data **aggregation** (e.g., more data through collaboration or consortia or data sharing, which also allows evidence synthesis through meta-analyses)		Exemplary data sharing projects/platforms: Many Labs Study 1; Many Labs Study 2; Many Babies Project; Psychological Science Accelerator; Play and Learning Across a Year Project	
				**Ethical concerns** (e.g., privacy, vulnerability, subject protection, local IRB-bound restrictions)		**Data anonymization** (e.g., use suggestions by the Declaration of Helsinki)		DeclarationofHelsinki	
						Share and consistent use of **standardized consent** material/wording		Open Brain Consent sample consent forms	
						Disclosure / restricted access if required			
				**Biological considerations** in DCN samples (e.g., distinct biology, reduced BOLD response, different physiology in MRI)		**Subject-specific solutions** (e.g., child-friendly head coils or response buttons, specific sequence, use highly engaging tasks)		CCHMC Pediatric Brain Templates; NIHPD pediatric atlases (4.5-18.5y); CCHMC Pediatric Brain Templates; Neurodevelopmental MRI Database	
**2. During & throughout data collection**	**FLEXIBILITY IN DATA COLLECTION STRATEGIES**								
				**Researchers degree of freedom I** (intransparent assessment choices, see Simmons et al., 2012, for a 21-word solution)		Increase **methods knowledge** across scientists (e.g., through hackathons and workshops)		Brainhack Global; Open Science MOOC; NeuroHackademy	
						**Teaching** reproducible research practices		Mozilla Open Leadership training; Framework for Open and Reproducible Research Training	
				**Variability & biases in study administration**		**Research project management tools:** standard training and protocol for data collection, use of logged lab notebooks, automation of processes		Human Connectome Project Protocols; Open Science Framework	
						**Standard operation procedure** (public registry possible; see Lin and Green, 2016)		Git version control (e.g., github.com)	
				**Flexible choice of measurements, assessments or procedures**		Policies / standardization / use of fixed protocols / age-adequate tool-& answer boxes			
				**Random choice of confounders**		**Code sharing**			
				**Data manipulation checks**					
						**Clear documentation** / detailed analysis plan / comprehensive data reporting		FAIR (Findable, Accessible, Interoperable and Re-usable) data principles; JoVE video methods journal; Databrary for sharing video data	
						**Preregistration**			
**3. Issues arising post data collection & consider throughout**	**ISSUES IN ANALYSES CHOICES & INTERPRETATION**								
						**Cross-validation** (e.g., k-fold or leave-one-out methods)			
				**Generalizability Robustness**		**Replication** (using alternative approaches or perform replication in alternative approaches)		Replication grant programs (e.g., NWO); Replication awards (e.g, OHBM Replication Award)	
						**Sensitivity analysis**			
				**Transparency** (inadequate access to materials, protocols, analysis scripts, and experimental data)		Make data **accessible** also furthering meta analytic options (e.g., sharing of raw data or statistical maps (i.e., fMRI), sharing code, sharing of analytical choices and references to the foundation for doing so) ideally in line with community standards		NeuroVault for sharing unthresholded statistical maps; OpenNeuro for sharing raw imaging data; Dataverse open source research data repository; Brain Imaging Data Structure	
						**Make studies **auditable****			
						Transparent, clear labelling of **confirmatory vs. exploratory** analyses		TOP (Transparency and Openness Promotion) guidelines	
	**Analytical Flexibility**								
				**Researchers degree of freedom II** (intransparent analysis choices)				Transparency Checklist (Azcel et al., 2019)	
				**hindsight bias** (consider results more likely after occurrence)		**disclosure / properly labeling** hypothesis-driven vs. confirmatory research			
				**p-hacking** (data manipulation to find p-significance)				Preregistration resources (may be embargoed/time-stamped amendments possible); The use of Preregistration Tools in Ongoing, Longitudinal Cohorts (SRCD 2019 Roundtable); Tools for Improving the Transparency and Replicability of Developmental Research (SRCD 2019 Workshop)	
				**p-harking** (hypothesizing after the results are known)					
				**t-harking** (transparently harking in the discussion section)					
				**s-harking** (secretly harking)		**Preregistration** (e.g., OSF; Aspredicted.org)			
				**cherry-picking** (running multiple tests and only reporting significant ones)		**Registered Reports** (review of study, methods, plan prior to data collection & independent of outcome)		Registered Reports resources (including list of journals using RRs); Secondary data preregistration template; fMRI Preregistration template (Flannery, 2018); List of neuroimaging preregistrations and registered reports examples	
				**Circularity** (e.g., circular data analysis)					
				Need for **multiple comparison correction**		***p*-curve analysis** (testing for replicability)			
				Random choice of **covariates**					
						**Specification curve analysis** (a.k.a. multiverse analyses; allows quantification and visualization of the stability of an observed effect across different models)		Specification curve analysis tutorial	
				**Overfitting**		**Cross-validation** (tests overfitting by using repeated selections of training/test subsets within data)			
				**Missing defaults** (e.g., templates or atlases in MRI research), representative comparison group (e.g., age, gender), more motion in neuroimaging studies		Subject-specific solutions (e.g., online motion control or protocols for motion control)		Framewise Integrated Real-time MRI Monitoring (FIRMM) software	
						Use of standardized toolboxes		Exemplary standardized analyses pipelines for MRI analyses: fMRIPrep preprocessing pipeline; LONI pipeline	
	**Software issues**								
				**Variability** due to differences in software versions and operating systems		Disclosure of relevant **software information** for any given analyses		Docker for containerizing software environments	
				**Software errors**		Making studies **re-executable** (e.g., Ghosh et al., 2017)			
	**Research Culture**								
				**Publication bias** (e.g., publication of positive findings only)		**Incentives** for publishing null-results / unbiased publication opportunities			
								Publishing null results:	
				**Bias-selection** and omission of null results (file drawer explanation: only positive results are published or publishing norms favoring novelty)		**Post data** for evaluation & independent review		Publishing null results: F1000 Research; bioRxiv preprint server; PsyArXiv preprints for psychological sciences	
						Less reliance on all-or-nothing **significance testing** (e.g., Wasserstein et al., 2019)			
						Use of **confidence intervals** (e.g., Cumming, 2013)			
						**Bayesian modeling** (e.g., Etz and Vandekerckhove, 2016)			
						**Behavior change** interventions (see Norris and O’Connor, 2019)			
				**Scientist's personal concerns** (e.g., risk of being scooped leading to non-transparent practices)		**Citizen science** (co-producing research aims)			
	**POPULATION SPECIFIC**								
				**Ethical reasons (e.g., that prohibit data sharing)**		**Anonymization** or sharing of group maps over individual data (i.e., T-maps)		De-identification Guidelines; Anonymisation Decision-making Framework	
						Follow **reporting guidelines**		EQUATOR reporting guidelines; COBIDAS checklist	
						**Maximize** participant's **contribution** (ethical benefit)			

## 2 Statistical power

Statistical power refers to the likelihood that a study will detect an effect when there is an effect to be detected. Power is determined by both the size of the effect in question and the study sample size, which is the number of participants or observations. The importance of statistical power cannot be underestimated. Especially when combined with publication bias - the tendency for only significant findings to be published, statistical power is intimately tied to replicability. There are different ways how power can influence replicability. First, underpowered studies that report very small effects need enormous replication samples to assess whether the effect is close enough to zero to be considered a null effect. Note that one way to circumvent this is the ‘small telescopes’ approach by Simonsohn (2015), which estimates whether the replication effect size is significantly smaller than an effect for which the original study had 33 % power to detect. Second, for replications to be informative, statistical power of the replication study needs to be high enough to be informative. It is therefore important to consider that underpowered studies can overestimate the effect size (and these overestimations are more likely to get published). When power calculations in a replication are based on such an inflated effect size, the actual replication power is much lower than proposed and results in an uninformative imprecise replication. In the context of developmental neuroimaging, we will first discuss issues related to sample size and effect sizes, before reviewing specific challenges of conducting small-sample size studies. We then discuss the opportunities – but also the challenges – for reproducibility and replicability that have arisen in recent years with the growing number of large, publicly available developmental cognitive neuroscience datasets.

### 2.1 Sample size

Adequate sample sizes are important for several reasons. As highlighted by Button et al. (2013), small samples reduce the chance of detecting a true effect, but it is less well appreciated that small samples also reduce the likelihood that a statistically significant result reflects a true effect or that small samples can yield exaggerated effects. The mechanism behind this latter bias is that measured effect sizes will have some variability due to sampling error (Szucs and Ioannidis, 2017). Studies with small samples will only be able to classify a true effect as significant on the occasional large overestimation of the effect size, meaning that when results of underpowered studies turn out to be significant, chances are high that the effect size is overestimated. In other words, small samples increase Type 2 errors (false negatives) and can lead to inflated Type 1 errors (false positives) in the literature when combined with the bias to publish studies with positive results. Button et al. (2013) used reported summary effects from 48 meta-analyses (covering 730 individual primary studies) in the field of neuroscience published in 2011 as estimates of the true effects and calculated the statistical power of each specific study included in the same meta-analyses. In this way, they empirically showed that the average statistical power was low in a range of subfields within neuroscience, including neuroimaging where they estimated the median statistical power of the studies at a meager 8 %. Later, Nord et al. (2017) reanalyzed data of the same sample of studies and found that the studies grouped together in several subcomponents of statistical power, including clusters of adequate or well-powered studies. But for the field of neuroimaging, the studies only grouped in two clusters, with the large majority showing relatively low statistical power and only a small group showing very high power. We speculate that developmental neuroimaging studies are overrepresented in the former group.

Adding to the bleak prospect of these findings, a recent empirical investigation reported low replicability rates of associations between gray matter volume and standard psychological measures in healthy adults, even in samples of around 200–300 participants (Masouleh et al., 2019). These authors tried to replicate brain-behavior associations within the same large sample by using multiple randomly generated subsamples of individuals, looking at different sizes of the initial ‘discovery’ samples and subsequent replication samples. They showed that brain-behavior associations for the psychological measures did not often overlap in the discovery and replication samples. Additionally, as the size of the subsamples decreased (from N = 326 to N = 138), the probability of finding spatially overlapping results across the whole brain also decreased (Masouleh et al., 2019). Using a similar approach for cortical thickness and resting state functional connectivity, a preprint by Marek et al. (2020) recently suggested that datasets in the order of N = 2000 are needed to reliably detect the small effect sizes of most brain-behavior associations.

For developmental neuroimaging, it is likely that the problem of low statistical power is even greater. First of all, children and adolescents are more difficult to recruit, and also to get high quality data from, than participants from, for instance, a young adult student population. Second, in order to study age-related differences and make inferences about development, participants at different ages are needed, increasing the required total sample size. Given time and financial constraints in research, these factors can lead to small samples and underpowered studies for developmental cognitive neuroscientists, which can exacerbate the problem of false positives in the literature when combined with publication bias. Here are some ways to reduce this problem:

#### 2.1.1 Sequential interim analyses

Prior to data collection, one of the steps that can be taken to reduce the problems associated with low statistical power is to preregister the study to reduce reporting biases, such as only reporting significant results or certain conditions in a given study (see section 3.4 for more detail). In this case, one can also choose to prespecify the use of sequential interim analyses during data collection. The use of sequential analyses allows researchers to perform a study with fewer participants because of the possibility to terminate data collection when a hypothesized result is significant (Lakens, 2014). First, the maximum sample size needed to detect your smallest effect size of interest at 80 % power is determined by a power analysis, as is typically done. However, with sequential interim analyses, researchers can evaluate the significance of an analysis with less than the optimal sample size so long as the analyses are adjusted for the false positive inflation that occurs due to multiple analyses. If the result is significant using criteria prespecified by the researcher under those more stringent conditions, then data collection can be stopped. Such a form of prespecified, transparent ‘data peeking’ is not commonly used in our field, but has recently gotten increased attention in infancy research (Schott et al., 2019). An example of a recent neuroimaging study using sequential analyses to examine the relationship between hippocampal volume and navigation ability can be found in Weisberg et al. (2019).

#### 2.1.2 Prevent participant dropout in longitudinal studies and address missing data

Especially in longitudinal studies it is critical to consider retention efforts and ways to keep participants engaged in the study. Retention efforts are important to be able to effectively measure change over time, but also need to be designed to prevent biases in who drops out of the study. If the characteristics of the children and families who repeatedly participate in research sessions differ significantly from those who dropout over time, this will bias the results observed in longitudinal research if not appropriately addressed (Telzer et al., 2018; Matta et al., 2018). Reported dropout rates in longitudinal neuroscience studies can range from 10 to 50 percent and might differ between age ranges (e.g., Peters and Crone, 2017; Rajagopal et al., 2014). Not uncommonly, dropout in developmental cognitive neuroscience studies that require an MRI scan is due to teenagers getting braces, in addition to the more widespread reasons for dropout in developmental studies: loss of contact with or loss of interest from the families involved. Therefore, it is important to proactively plan to account for dropout due to predictable reasons (e.g., braces during early adolescence) and to make it a great experience for young participants and their families to take part in the study (Raschle et al., 2012). Fortunately, many developmental cognitive neuroscience labs do this very well, and we encourage research groups to share their tips and tricks for this practical side of the data collection that can facilitate participant recruitment and high retention rates in longitudinal studies. Formats that may be used to share more practical information on study conduction are for example video documentations as may be done through the journal of visualized experiments (https://www.jove.com/: for an exemplary pediatric neuroimaging protocol see Raschle et al., 2009), or the online platform databrary (https://nyu.databrary.org/). The Adolescent Brain Cognitive Development (ABCD; https://abcdstudy.org) study that is currently following 11,875 children for 10 years, has described their efforts to ensure retention in a recent article (Feldstein Ewing et al., 2018). Their efforts focus on building rapport through positive, culturally sensitive interactions with participants and their families, conveying the message to families that their efforts to participate are highly valued. But even if participants are willing to participate in subsequent study sessions, data might be lost due to issues such as in-scanner movement. Our section on data collection (section 3.1) and data quality (section 3.2) describes ways to ensure high data quality in younger samples. Finally, it is not always possible to prevent participant drop-out—families will move and some families might encounter a sudden change in household stability. This is why it is crucial to think carefully about missing data in a longitudinal study and model data using the least restrictive assumptions about missingness (for an extensive review of handling missing data in longitudinal studies, please see Matta et al., 2018).

### 2.2 The importance of effect sizes

The focus on significant results in small samples, partly because such positive results get published more often, is one of the reasons why many published results turn out to be non-replicable. To overcome the overreliance on binary decision rules (e.g., significant versus nonsignificant in the currently dominant frequentist framework), researchers might focus more on reporting effect sizes (a description of the magnitude of an effect; Reddan et al., 2017). Reporting effect sizes and putting them into context, is something that all studies can do to describe the relevance of a particular finding, and will also aid future power calculations. Putting effect sizes in context can take the form of addressing how the observed effect compares to other variables in the present study, or how the observed effect compares to what has been observed in other studies. To give a few examples: in a longitudinal developmental cognitive neuroscience study, one could report a significant negative linear relationship between cortical thickness and age during adolescence. But reporting the average annual percent decrease in cortical thickness would be one way to illustrate the effect size in an understandable and easily comparable way. By doing so, readers can see how the annual decrease in cortical thickness observed during adolescence compares to what is observed in the aging literature, or to the impact of, for example, training interventions on cortical thickness. To take another example, reporting how correlations in spontaneous BOLD fluctuations, measured in resting-state functional MRI, relate to age can be put into context by comparing them to the effect sizes reported in studies of mental health or behavior.

Statistical power is also a product of the effect size, which makes this an important measure for power calculations. Effect sizes can vary substantially in developmental cognitive neuroscience, depending on the topic of interest. A general recommendation is to design a study around an *a priori* power calculation drawing from the existing literature (e.g., using tools such as http://www.neuropowertools.org). However, in doing so one must take into account that due to reporting bias in the present literature, reported effect sizes are often inflated (Cremers et al., 2017). While power calculation is not as straightforward for longitudinal study designs, simulation approaches can be adopted in open-source software packages available in R (e.g., powerlmm; simsem). When there is limited data regarding what effect size could be expected for a given analysis, researchers can instead identify a smallest effect size of interest (SESOI; Lakens et al., 2018). In the following sections, we discuss challenges and solutions related to conducting studies on small or moderate effect sizes, and separately for small sample studies and large studies.

### 2.3 How to value small sample studies?

For reasons such as the costs associated with recruiting and testing developmental samples, it can be difficult to obtain sample sizes that yield sufficient statistical power when the effect size is small to medium at best. However, trying to publish a developmental neuroimaging study with a small sample of participants is becoming increasingly more difficult. But does this mean that we should stop performing small sample studies, altogether? We believe it is still worth considering small sample studies, at least in some situations. One example is that studies with small samples can have value by proof of concept or conceptual innovation. Another example is that small sample studies can have value by addressing understudied research questions or populations. Below, we consider recommendations on how these small sample investigations can be done in a meaningful way.

#### 2.3.1 Cumulative science from small samples

The sample size needed for a well-powered study is dependent on multiple factors such as the presumed effect size and study design. But in general, the typical sample sizes of 20–30 participants are usually underpowered to detect small to medium within-subject effects (Cremers et al., 2017; Poldrack et al., 2017; Turner et al., 2018). For detecting between-subject effects of the average size reported (e.g., Cohen’s *d* of 0.2; see Gignac and Szodorai, 2016), even larger sample sizes are needed. For correlational analysis designs it has been suggested that sample sizes of at least 150–250 participants are needed in order to ensure stable findings in the context of behavioral or questionnaire studies (Schönbrodt and Perugini, 2013). However, a sample size in that range is often not feasible for smaller developmental cognitive neuroscience laboratories or for researchers studying specific low prevalence clinical conditions. This should not mean that work on smaller, challenging-to-recruit samples should be abandoned. For one, the cumulative output from many underpowered studies may be converged in order to obtain a reliable conclusion, for example through meta-analytic approaches. Indeed, a meta-analysis of five geographically or in any other way diverse studies with N = 20 will lead to more generalizable conclusions than one N = 100 study from a single subpopulation. However, for this to be true, each individual study needs to be up to the highest standards of transparency and sharing of materials to allow a convergence of the data to ensure reproducibility. Furthermore, meta-analytic approaches are not invulnerable to the problem of publication bias. If meta-analytic procedures are built upon a biased selection of published findings, and if they cannot include null-findings within their models, then the resulting output is similarly problematic. As a feasible solution to ensure an unbiased study report, steps that can be taken before data collection are preregistration or submitting a Registered Report. Especially Registered Reports (preregistrations submitted to a journal to be reviewed before data collection or analysis) guard against publication bias because the acceptance of the article will be independent of the study outcome (see section 3.4). The integrated peer-review feedback on the methods section of the proposed study should also positively impact the quality of the methods employed; altogether fostering reproducibility. After data collection, sharing results should include the provision of unthresholded statistical imaging maps to facilitate future meta-analyses, which can for example be done through NeuroVault (www.neurovault.org; Gorgolewski et al., 2015).

After data collection, several steps at the level of statistical analyses (which should also be considered before data collection when designing a study) can be taken to increase the replicability and validity of work with smaller samples. For one, given the lower statistical power of studies with smaller samples, it is advisable to limit the number of hypotheses tested, and thus reduce the number of analyses conducted. This will limit the complexity of the statistical analyses and the need for or degree of adjustment for multiple comparisons. For neuroimaging research, limiting the number of analyses can be achieved in several ways, from the kind of scan sequences obtained to the regions of the brain examined. However, this necessitates a strong theoretical basis for selecting a specific imaging modality or region of the brain to examine, which might not be feasible for research lines impacted by publication bias. In that case, regions of interest are affected by publication bias because significant effects in regions of interest are more likely to be reported than nonsignificant effects. Without preregistration of all *a priori* regions of interest and all subsequent null findings, it is hard to consider the strength of the evidence for a given region. This is further complicated because heterogeneity in spatial location and cluster size across studies for regions with the same label lead to imprecise replications of effects (Hong et al., 2019). One way to specify regions of interests less affected by publication bias is the use of coordinate-based meta-analysis. Another way is the use of parcellations in which brain regions are divided based on structural or functional connectivity-based properties (Craddock et al., 2012; Eickhoff et al., 2018; Gordon et al., 2016). To ensure transparency, *a priori* selections can be logged through preregistration. Another example of limiting the complexity of a developmental cognitive neuroscience analysis would be to focus on effects for which *a priori* power was calculated. In practice, this means that especially in smaller samples, researchers should avoid analyses with ever smaller subgroups or post hoc investigation of complex interaction effects. We are aware that this might put early career researchers and others with less resources at a disadvantage, as they are under more pressure to make the most out of smaller studies. Reviewers and editors can support authors who clearly acknowledge the limitations of their samples and analyses, by not letting this transparency affect the chances of acceptance of such a paper. It is also worth considering that taking steps to reduce the number of false positives in the literature will make it less likely that early career researchers will waste time and resources trying to build upon flawed results.

#### 2.3.2 More data from small samples

It is also important to point out that a small sample of subjects does not have to mean a small sample in terms of data points. In relation to statistical power, the number of measurements is a particularly crucial factor (Smith and Little, 2018). This is also true for task-based functional neuroimaging studies, in which longer task duration increases the accuracy to detect effects due to increased temporal signal to noise ratio (Murphy et al., 2007). More so, under optimal noise conditions with large amounts of individual functional magnetic resonance imaging (fMRI) data, task-related activity can be detected in the majority of the brain (Gonzalez-Castillo et al., 2012). Even with modest sample sizes of around 20 participants, the replicability of results increases when more data is collected within individuals on the same task (Nee, 2019). This is because the amount of noise is reduced not only by decreasing between-subject variance (by collecting data from more individuals) but also by decreasing within-subject variance (by collecting more data per individual). For example, when replicability is operationalized as the correlation between voxels, clusters, or peaks in two or more studies with different samples using the same methods (cf., Turner et al., 2018), the correlations will become stronger when the signal to noise ratio is boosted. This does not mean that scanning just a few participants extremely long would equal scanning many participants very shortly: at some point the gain from decreasing within-subject variance will lead to little improvement in power, meaning that power can then only be improved by decreasing between-subject variance through increasing the sample size (Mumford and Nichols, 2008).

There are several examples of highly informative cognitive neuroscience investigations that deeply phenotype only a single or few participants (Poldrack et al., 2015; Choe et al., 2015; Filevich et al., 2017). Following the pioneering work of the MyConnectome project by Poldrack et al. (2015), studies by the Midnight Scan Club are based on the data of only ten individuals (Gordon et al., 2017). This dataset includes 10 h of task-based and resting-state fMRI data per participant, allowing individual-specific characterization of brain functioning and precise study of the different effects of individual, time, and task variability (Gratton et al., 2018). These and other studies (e.g., Filevich et al., 2017) demonstrate that high sampling rates can solve some of the power issues related to small samples. Analogous to the Midnight Scan Club, Marek et al. (2018) managed to collect 6 h of resting-state fMRI data during 12 sessions in one 9 year old boy. However, highly sampling young participants, as would be the goal in developmental cognitive neuroscience investigations, warrants special consideration (e.g., feasibility or ethical concerns). Furthermore, deep-phenotyping does not reduce costs related to scanning on multiple occasions, nor is it feasible for many cognitive tasks to be sampled on such a frequency. Additionally, small samples, often with tightly controlled demographics, cannot inform about population variability. This means that such studies remain inherently limited when it comes to generalization to the wider population, and should be interpreted accordingly (see LeWinn et al., 2017 for how non-representative samples can affect results in neuroimaging studies). However, despite such caveats, within the limits of ethical possibilities with young participants, increasing the amount of within-subject data by using fewer but longer tasks within sessions, or by following up smaller cohorts more extensively or for a longer time, will increase power within subjects (see Vidal Bustamante et al. (2020) for an example of a study in which adolescents partake in monthly MRI scans, surveys and interviews).

#### 2.3.3 More reliable data from (sm)all samples

For smaller sample studies, it is of the utmost importance to reduce sampling error on as many levels as possible. In the context of cognitive development, it is necessary to make sure the behavior on experimental paradigms is robust and reliable. High test-retest reliability - meaning the paradigm produces consistent results each time it is used (Herting et al., 2018a) - should therefore be established before a developmental study is performed (for both small and large samples). Psychometric properties such as reliability also need to be reported post hoc, since these are mainly properties of the test in a particular setting and sample (Cooper et al., 2017; Parsons et al., 2019b). Establishing reliability is important for several reasons: 1) it provides an estimate of how much the scores are affected by random measurement error, which in turn is a prerequisite of the validity of the results (i.e., does the test measure what it is supposed to measure). 2) If we want to relate the scores with other measures such as imaging data, low reliability in one of the measures compromises the correlation between the two measures. 3) With lower reliability, statistical power to detect meaningful relationships decreases (Hedge et al., 2018; Parsons et al., 2019b). 4) Many experimental tasks were designed to produce low between-person variability, making them less reliable for studying individual differences (Hedge et al., 2018).

In addition, in the case of developmental neuroimaging, one must go beyond reliability of behavioral measures, but should also establish test-retest reliability for functional activity. Test-retest reliability of BOLD responses is not regularly reported, but several studies have shown poor to fair results for some basic tasks (Plichta et al., 2012; van den Bulk et al., 2013). For more complex tasks, the underlying cognitive processes elicited should be reliable as well, given that many more complex experimental tasks can be solved relying on different cognitive processes. For instance, it is known that across development children and adolescents start making use of more complex decision rules (Jansen et al., 2012), and that these decision rules are associated with different patterns of neural activity (van Duijvenvoorde et al., 2016). Such variability in cognitive strategies may not be visible on the behavioral level, but will have a negative effect on the reliability of the neural signals. More so, poor test-retest reliability for task fMRI might partly stem from the use of tasks with poor psychometric validity. Unfortunately, psychometric properties of computerized tasks used in experimental psychology and cognitive neuroscience are underdeveloped and underreported, compared to self-report questionnaires (Enkavi et al., 2019; Parsons et al., 2019b).

In sum, especially in the case of smaller samples, replicability might be increased by using relatively simple and reliable tasks with many trials. Naturally, at some point, unrestrained increases in the length of paradigms might backfire (e.g., attention to task will fade, motion will increase), especially in younger participants. One option might be to increase total scan time by collecting more runs that are slightly shorter. For instance, Alexander et al. (2017) reported more motion in the second half of a resting state block than during the first half and subsequently split the block into two for subsequent data collection. The optimal strategy for increased within-subject sampling in developmental studies remains an empirical question. It might therefore be good to point out that reliability also depends on factors related to analytic strategies used after data collection. Optimizing data analysis for these purposes, for instance by the choice of filter selection and accounting for trial-by-trial variability, could help to lower the minimum data required per individual to obtain reliable measures (Rouder and Haaf, 2019; Shirer et al., 2015; Zuo et al., 2019).

#### 2.3.4 Collaboration and replication

Another option for increasing the value of small samples is to work collaboratively across multiple groups, either by combining samples to increase total sample sizes or by repeating the analyses across independent replication samples. One can also obtain an independent replication sample from the increasing number of open datasets available (see section 2.4). Collaborative efforts can consist of post-hoc data pooling and analyses, as has for example been done within the 1000 Functional Connectomes Project (Biswal et al., 2010) and the ENIGMA consortium (P. M. Thompson et al., 2020a), or even with longitudinal developmental samples (Herting et al., 2018b; Mills et al., 2016; Tamnes et al., 2017). Such collaborations can also be conducted in a more pre-planned fashion. For instance, to make your own data more usable for the accumulation of data across sites, it is important to see if standardized procedures exist for the sequences planned for your study (e.g., the Human Connectome Project in Development sequence for resting state fMRI; Harms et al., 2018). These standards might sometimes conflict with the goals of a specific study, say when interested in optimizing data acquisition for a particular brain region. Of course, in such cases it could be better to deviate from standardized procedures. But in general, well-tested acquisition standards such as used in the Human Connectome Project would aid most researchers in collecting very high quality data ((Glasser et al., 2016) Harms et al., 2018). With increased adoption of standards, such data will also become easier to harmonize with data from other studies.

The ManyBabies Project is a collaborative project example that focuses specifically on assessing the “replicability, generalizability, and robustness of key findings in infancy,” by combining data collection across different laboratories (https://manybabies.github.io/). In contrast with the Reproducibility Project (Open Science Collaboration, 2015), all participating labs jointly set up the same replication study with the goal of standardizing the experimental setup where possible and carefully documenting deviations from these standards (Frank et al., 2017). Such an effort not only increases statistical power, but also gives more insight into the replicability and robustness of specific phenomena, including important insights into how these may vary across cultures and measurement methods. For example, within the first ManyBabies study three different paradigms for measuring infant preferences (habituation, headturn preference, and eye-tracking) were used at different laboratories, in which the headturn preference led to the strongest effects (ManyBabies Consortium, 2020). A similar project within developmental neuroimaging could start with harmonizing acquisition of resting-state fMRI and T1-weighted scans and agreeing on a certain set of behavioral measures that can be collected alongside ongoing or planned studies. In this way, the number of participants needed to study individual differences and brain-behavior correlations could be obtained through an international, multisite collaboration. A more far-reaching collaboration resembling the ManyBabies Project could be to coordinate collection of one or more specific fMRI or EEG tasks at multiple sites to replicate key developmental cognitive neuroscience findings. This would also provide an opportunity to collaboratively undertake a preregistered, high-powered investigation to test highly influential but debated theories such as imbalance models of adolescent development (e.g., Casey, 2015; Pfeifer and Allen, 2016).

### 2.4 New opportunities through shared data and data sharing

Increasingly, developmental cognitive neuroscience datasets are openly available. These range from small lab-specific studies, to large multi-site or international projects. Such open datasets not only provide new opportunities for researchers with limited financial resources, but can also be used to supplement the analyses of locally collected datasets. For example, exploratory analyses can be conducted on large open datasets to narrow down more specific hypotheses to be tested on smaller samples. Open datasets can also be used to replicate hypothesis-driven work, and test for greater generalizability of findings when the variables of interest are similar but slightly different. Open datasets can also be used to prevent double-dipping, for example by defining regions of interest related to a given process in one dataset, and testing for brain-behavior correlations in a separate dataset.

Access to openly available datasets can be established in a number of ways, here briefly outlined in three broad categories: large repositories, field or modality-specific repositories, and idiosyncratic data-sharing. Note that using these datasets should ideally be considered before collecting new data, which provides the opportunity to align one’s own study protocol with previous work. This can also help with planning what unique data to collect in a single lab study that could complement data available in large scale projects. Before data collection, it is also very important to consider the possibilities (and the obligations for an increasing number of funding agencies) of sharing the data to be collected. This can range from adapting informed consent information to preparing a data management plan to make the data human- and machine-readable according to recognized standards (e.g., FAIR principles, see Wilkinson et al., 2016). After data collection, open datasets can be used for cross-validation to test the generalizability of results in a specific sample (see also section 2.6).

With increasing frequency, large funding bodies have expanded and improved online archiving of neuroimaging data, including the National Institute of Mental Health Data Archive (NDA; https://nda.nih.gov), and the database of Genotypes and Phenotypes (dbGaP; https://www.ncbi.nlm.nih.gov/gap/). Within these large data archives, researchers can request access to lab-specific datasets (e.g., The Philadelphia Neurodevelopmental Cohort), as well as access to large multi-site initiatives like the ABCD study. Researchers can also contribute their own data to these larger repositories, and several funding mechanisms (e.g., Research Domain Criteria, RDoC) mandate that researchers upload their data in regular intervals. The NIMH allows for researchers who are required to share data to apply for supplemental funds which cover the associated work required for making data accessible. Thereby, the funders help to ensure that scientists comply with standardized data storage and structures, while recognizing that these are tasks requiring substantial time and skill. While these large repositories are a centralized resource that can allow researchers to access data to answer theoretical and methodological hypotheses, the format of the data in such large repositories can be inflexible and may not be as well-suited to neuroimaging data.

Data repositories built specifically for hosting neuroimaging data are becoming increasingly popular. These include NeuroVault (https://neurovault.org; Gorgolewski et al., 2015), OpenNeuro (https://openneuro.org; Poldrack and Gorgolewski, 2017), the Collaborative Informatics and Neuroimaging Suite (COINS; https://coins.trendscenter.org; Scott et al., 2011), the NITRC Image Repository (https://www.nitrc.org/; Kennedy et al., 2016) and the International Neuroimaging Data-sharing Initiative (INDI; http://fcon_1000.projects.nitrc.org; Mennes et al., 2013). These are open for researchers to utilize when sharing their own data, and host both small and large-scale studies, including the Child Mind Institute Healthy Brain Network study (Alexander et al., 2017), and the Nathan Kline Institute Rockland Sample (Nooner et al., 2012). These data repositories are built to handle neuroimaging data, and can more easily integrate evolving neuroimaging standards. For example, the OpenNeuro website mandates data to be uploaded using the Brain Imaging Data Structure (BIDS) standard (Gorgolewski et al., 2016), which then can be processed online with BIDS Apps (Gorgolewski et al., 2017).

Idiosyncratic methods of sharing smaller, lab-specific, data with the broader community might result in less utilization of the shared datasets. It is possible that researchers are only aware of these datasets through the empirical paper associated with the study, and the database hosting the data could range from the journal publishing the paper, to databases established for a given research field (e.g., OpenNeuro), or more general data repositories (e.g., Figshare, Datadryad). However, making lab-specific datasets available can help further efforts to answer methodological and theoretical questions, and these datasets can be pooled with others with similar measures (e.g., brain structure) to assess replicability. Further, making lab-specific datasets openly available benefits the broader ecosystem by providing a citable reference for the early career researchers who made it accessible.

### 2.5 Reproducibility and replicability in the era of big data

The sample sizes in the largest neuroimaging studies, including the largest developmental neuroimaging studies, are rapidly increasing. This is clearly a great improvement in the field. Large studies yield high statistical power, likely leading to more precise estimates and lower Type 2 error rates (i.e., less false negatives). However, critically considering the power of these studies paired with an overemphasis on statistical significance, increases the risk of over-selling small effect sizes. Furthermore, large and rich datasets offer a lot of flexibility at all stages of the research process. Both issues represent novel, though increasingly important, challenges in the field of developmental cognitive neuroscience.

While making data accessible is a major step forward, it can also open up the possibility for counterproductive data mining and dissemination of false positives. Furthermore, with a large dataset, traditional statistical approaches emphasizing null-hypothesis testing may yield findings that are statistically significant, but lack practical significance. Questionable research practices, such as conducting many tests but only reporting the significant ones (*p*-hacking or selective reporting) and hypothesizing after the results are known (HARKing), exacerbate these problems and hinder progress towards the development of meaningful insights into human development and its implications for mental health and well-being. High standards of transparency in data reporting could reduce the risk of such problems. This may include preregistration or Registered Reports of analyses conducted on pre-existing datasets, developing and sharing reproducible code, and using holdout samples to validate model generalizability (see also Weston et al., 2019, for a discussion).

To describe one of these examples, reproducibility may be increased when analysis scripts are shared, particularly when several researchers utilize the same open dataset. As the data are already available to the broader community, the burden to collect and share data is no longer placed on the individual researcher, and effort can be channeled into creating a well-documented analytic script. Given its availability, it does become likely that multiple researchers ask the same question using the same dataset. In the best-case scenario, multiple papers might then be published with similar results at the same time; allowing an excellent opportunity to evaluate the robustness of a given study result. However, a valid concern may be that one study is published while another is being reviewed. But as mentioned in Laine (2017), it may be equally as likely that competing research teams end up collaborating on similar questions or avoid too much overlap from the beginning. It is possible, and has previously been demonstrated in social psychology (Silberzahn et al., 2018), that different teams might ask the same question of the same dataset and produce different results. Recently, results were published of a similar effort of 70 teams analyzing the same fMRI dataset, showing large variability in analytic strategies and results (Botvinik-Nezer et al., 2020; https://www.narps.info). Methods such as specification curve analysis or multiverse analysis have been proposed as one way to address the possibility of multiple analytic approaches generating different findings, detailed below in Section 3.3.

Another way that we can proactively address the possibility of differential findings obtained across groups is to support the publication of meta-analyses or systematic summaries of findings generated from the same large-scale dataset regularly. Such overviews of tests run on the same dataset can help to get better insight in the robustness of the research findings. For example, when independent groups have looked at the relation between brain structure and substance use using different processing pipelines, the strength of the evidence can be considered by comparing these results. Another problem that can be addressed using regular meta-analyses is the increasing false positive rate when multiple researchers run similar, confirmatory statistical tests on the same open dataset. False positive rates will increase if no correction for multiple comparisons is applied for tests that belong to the same ‘statistical family’ but are being conducted by different researchers, and at different times W. H. Thompson et al., 2020. When the number of preceding tests is known, researchers can use this information to correct for new comparisons they are about to make, alternatively some form of correction could be applied retrospectively (see W. H. Thompson et al., 2020b, for an in-depth discussion on ‘dataset decay’ with re-using open datasets).

### 2.6 The danger of overfitting and how to reach generalizability

One way of understanding the reports of high effects sizes in small samples studies is that they are the result of overfitting of a specific statistical model (Yarkoni and Westfall, 2017). Given the flexibility researchers have when analyzing their data it is possible that a specific model (or set of predictors) result in very high effect sizes. This is even more likely when there are many more predictors than participants in the study. Within neuroimaging research this is something that quickly happens as a result of the large number of voxels representing one brain volume. A model that is overfitting is basically fitting noise, and thus it will have very little predictive value and a small chance being replicated. One benefit of large samples of subjects is that they provide opportunities to prevent overfitting by means of cross-validation (i.e., k-fold or leave-one-subject-out cross-validation; Browne, 2000), ultimately allowing for more robust results. Simply put, the data set is split into a training set and a validation (or testing) set. The goal of cross-validation is to test the model's ability to predict new data from the validation set based on its fit of the training set.

Although cross-validation can easily be used in combination with more classic *confirmatory* analyses to test the generalizability of an a priori determined statistical model, it is more often used in *exploratory* predictive modeling and model selection. Indeed, the use of machine-learning methods to predict behavior from brain measures has become increasingly common, and is an emerging technique in (developmental) cognitive neuroscience (for an overview see Rosenberg et al., 2018; or Yarkoni and Westfall, 2017). Predictive modeling is specifically of interest when working with large longitudinal datasets generated by consortia (e.g., ABCD or IMAGEN). These datasets often contain many participants but also commonly include far more predictors (e.g., questionnaire items, brain parcels or voxels). For this type of data, the predictive analyses used are often a form of regularized regression (e.g., Lasso or elastic net), in which initially all available, or interesting, regressors are used in order to predict a single outcome. A relevant developmental example is the study by Whelan et al. (2014), which investigated a sample of 692 adolescents to predict future alcohol misuse based on brain structure and function, personality, cognitive abilities, environmental factors, life experiences, and a set of candidate genes. Using elastic net regression techniques in combination with nested cross-validation this study found that from all predictors, life history, personality, and brain variables were the most predictive of future binge drinking.

### 2.7 Interim summary

Statistical power is of utmost importance for reproducible and replicable results. One way to ensure adequate statistical power is to increase sample sizes based on *a priori* power calculations (while accounting for expected dropout), and at the same time decreasing within-subject variability by using more intensive, reliable measures. The value of studies with smaller sample sizes can be increased by high standards of transparency and sharing of materials in order to build cumulative results from several smaller sample studies. In addition, more and more opportunities are arising to share data and use data shared by others to complement and accumulate results of smaller studies. When adequate and transparent methods are used, the future of the field will likely be shaped by an informative mix of results from smaller, but diverse and idiosyncratic samples, and large-scale openly available samples. In the following, we discuss the challenges and opportunities related to flexibility and transparency in both smaller and larger samples in more detail.

## 3 Flexibility and transparency in data collection and data analysis

In light of the increasing sample sizes and richness of datasets in developmental cognitive neuroscience available, a critical challenge to reproducibility and replicability is the amount of flexibility researchers have in data collection, analysis and reporting (Simmons et al., 2011). The amount of flexibility is even intensified in the case of high-dimensional neuroimaging datasets (Carp, 2012; Botvinik-Nezer et al., 2020). On top of this, in developmental studies many choices have to be made about age groupings, ways of measuring development or puberty, whilst a longitudinal component adds another level of complexity. In the following, we discuss some examples of designing and reporting studies that lead to increased transparency to aid reproducibility and replicability. First we discuss how data collection strategies can increase replicability, followed by the importance of conducting and transparently reporting quality control in developmental neuroimaging. Next, we discuss specification curve analysis as a method in which a multitude of possible analyses are transparently reported to establish the robustness of the findings. Finally, we discuss how preregistration of both small- and large-scale studies can aid methodological rigor in the field.

### 3.1 Increasing transparency in data collection strategies

Practical and technical challenges have long restricted the use of (f)MRI at younger ages such as infancy or early childhood (see Raschle et al., 2012), whereas the adolescent period has now been studied extensively for over two decades. Fortunately, technical and methodological advances allow researchers to conduct neuroimaging studies in a shorter amount of time, with higher precision and more options to correct for shortcomings associated with pediatric neuroimaging (e.g., motion). Such technical advances thus make MRI more suitable to be applied in children from a very young age on, opening possibilities to study brain development over a much larger course of development from birth to adulthood. One downside is that the replicability of this work can be impacted by the variability in data collection and processing strategies when scanning younger adolescents and children. It is therefore necessary to transparently report how data was collected and handled to aid replication and generalizability. The publication of protocols can be helpful because they provide standardized methods that allow replication. For example, there is an increasing number of publications, including applied protocols and guidelines, providing examples of age-appropriate and child-friendly neuroimaging techniques that can be used to increase the number of included participants and increase the likelihood to obtain meaningful data (e.g., de Bie et al., 2010; Pua et al., 2019; Raschle et al., 2009).

A focus on obtaining high quality, less motion-prone, MRI data can also mean reconsidering the kind of data we collect. One example is the use of engaging stimuli sets such as movies, as a way to create a positive research experience to get high quality data from young participants. Especially in younger children, movies provide an improvement in head motion during fMRI scanning relative to task and resting-state scans (Vanderwal et al., 2019). Movies might be used to probe activation in response to a particular psychological event in an engaging, task-free manner. For example, a study by Richardson and colleagues used the short Pixar film ‘Partly Cloudy’ to assess functional activation in Theory of Mind and pain empathy networks in children aged 3–12 years (Richardson et al., 2018). In the context of the current review it is mentionable that Richardson (2019) subsequently used a publicly available dataset (Healthy Brain Network; Alexander et al., 2017) in which participants watched a different movie to replicate this finding. This work shows the potential of movie-viewing paradigms for developmental cognitive neuroscience, even with different movies employed across multiple samples. Apart from using movies as a stimulus of interest, movie viewing can also be used to reduce head motion during structural MRI scans in younger children (Greene et al., 2018).

Another choice to be made before collecting data is the use of a longitudinal or cross-sectional design. This choice is of course dependent on the available resources, with longitudinal research much less feasible for early career researchers. Notably, early career researchers might not even be able to collect and implement longitudinal datasets during their appointment. Although the majority of developmental cognitive neuroscience studies to date are based on cross-sectional study designs, these studies are limited in their ability to inform about developmental trajectories and individual change over time (Crone and Elzinga, 2015). From the perspective of reproducibility and replicability, it is also important to consider that longitudinal studies have much higher power to detect differences in measures such as brain volume that vary extensively between individuals (Steen et al., 2007). This is because in a longitudinal study, only measurement precision affects the required sample size, whereas in cross-sectional studies both measurement precision and natural variation of brain sizes between participants affect the required sample size. For example, in an empirical demonstration of this phenomenon by Steen et al. (2007), it was found that a cross-sectional study of grey matter volume requires 9-fold more participants than a longitudinal study. Thus, when possible, using longitudinal designs is important not only for drawing developmental inferences but also to increase power. Longitudinal designs also bring other challenges, such as retention problems (see section 2.1). Another potential difficulty is the differentiation of change and error in longitudinal modelling, as changes might reflect a combination of low measurement reliability and true developmental change (see Herting et al., 2018a, for an excellent discussion of this topic). In all instances, transparently reporting choices made in data collection and acknowledging limitations of cross-sectional analyses are vital for the appropriate interpretation of developmental studies.

### 3.2 Increasing methodological transparency and quality control

For many of the methodological issues outlined in the previous sections, there are multiple possible strategies, all with their own pros and cons. Hence, increasing reproducibility and replicability is not only a matter of what methods are being used, but much more about how accurate and transparent these methods are being reported. This could also include ways to implement and report quality control of neuroimaging measures. One major issue for developmental cognitive neuroscience is the fact that neuroimaging data quality is negatively impacted by in-scanner motion, which impacts measures of brain structure (Blumenthal et al., 2002; Ling et al., 2012; Reuter et al., 2015) and function (Power et al., 2012; Van Dijk et al., 2012). More problematic is the fact that motion is related to age: many studies have shown that younger children move more, resulting in lower scan quality that affect estimates of interest (Alexander-Bloch et al., 2016; Klapwijk et al., 2019; Rosen et al., 2017; Satterthwaite et al., 2012; White et al., 2018). The way quality control methods deal with motion artifacts can eventually impact study results. In one study that investigated the effect of stringent versus lenient quality control on developmental trajectories of cortical thickness, many nonlinear developmental patterns disappeared when lower quality data was excluded (Ducharme et al., 2016). Similarly, in case-control studies more strict quality control can lead to less widespread and less attenuated group differences. This was demonstrated in a recent multicenter study that investigated cortical thickness and surface area in autism spectrum disorders, in which 1818 from the initial dataset of 3145 participants were excluded after stringent quality control (Bedford et al., 2019). These and other studies underline the importance of quality control methods for neuroimaging studies, but there are currently no agreed standards for what counts as excessive motion or when to consider a scan unusable (Gilmore et al., 2019; Vijayakumar et al., 2018). It is therefore crucial to use strategies to minimize the existence and impact of motion and at the same time increase the transparency and reporting of these strategies in manuscripts.

Before and during data collection, there are different options to consider that can reduce the amount of in-scanner motion. Some of these strategies to improve data quality can be nontechnical, such as providing mock scanner training or using tape on the participant’s forehead to provide tactile feedback during actual scanning (de Bie et al., 2010; Krause et al., 2019). Many researchers also use foam paddings to stabilize the head and reduce the possibility for motion. A more intensive and expensive, but probably effective method is the use of 3D-printed, individualized custom head molds to restrain the head from moving (the current cost of $100−150 per mold would still be substantially lower than an hour of scanning lost to motion). These custom head molds have been shown to significantly reduce motion and increase data quality during resting-state fMRI in a sample of 7−28 year old participants (Power et al., 2019). Importantly, these authors report that participants, including children with and without autism, find these molds comfortable to wear, suggesting it does not form an additional burden when being scanned. Another recent paper found that molds were not more effective than tape on the forehead during a movie-viewing task in an adult sample (Jolly et al., 2020), stressing the need for more systematic work to establish the effectiveness of head molds.

With the availability of methods to monitor real-time motion during scanning, opportunities have arisen to prospectively correct for motion, to provide real-time feedback to participants or to restart a low-quality scan sequence. For structural MRI, methods are available to correct for head motion by keeping track of the current and predicted position of the participant within the scanner and use selective reacquisition when needed (Brown et al., 2010; Tisdall et al., 2012; White et al., 2010). For resting state and task-related functional MRI, Dosenbach et al. (2017) developed software called FIRMM (fMRI Integrated Real-time Motion Monitor; https://firmm.readthedocs.io/) that can be used to monitor head motion during scanning. This information can be used to scan each participant until the desired amount of low-movement data has been collected or to provide real-time visual motion feedback that can subsequently reduce head motion (Dosenbach et al., 2017; Greene et al., 2018).

Overall, it is important to use quality control methods to establish which scans are of usable quality after neuroimaging data collection. More so, the methods used for making decisions about scan quality should be reported transparently. As has been noted by (Backhausen et al., 2016), many studies only report very briefly that quality control was performed without much detail. With more details, for example by using established algorithms or links to protocols used for visual inspection, the ability to recreate study results increases. Some form of preliminary quality control is commonly implemented by most research teams, using visual or quantitative checks to detect severe motion. For example, functional MRI studies can use a certain threshold of the mean volume-to-volume displacement (framewise displacement) to exclude participants (Parkes et al., 2018). Likewise, standardized preprocessing pipelines may be used that provide extensive individual and group level summary reports of data quality, such as fMRIprep for functional MRI (https://fmriprep.readthedocs.io; Esteban et al., 2019) and QSIprep for diffusion weighted MRI (https://qsiprep.readthedocs.io). For extensive quality assessments of raw structural and functional MRI data, software like MRIQC (https://mriqc.readthedocs.io; Esteban et al., 2017) and LONI QC (https://qc.loni.usc.edu; Kim et al., 2019) provide a list of different image quality metrics that can be used to flag low quality scans. Decisions about the quality of processed structural image data can further be aided by the use of machine-learning output probability scores, as for instance implemented in the Qoala-T tool for FreeSurfer segmentations (Klapwijk et al., 2019). These software packages can help to reduce the subjective process of visual quality inspection by providing quantitative measures to compare data quality across studies, ultimately leading to more transparent standards for usable and unusable data quality. With increasing sample sizes, automated quality control methods also become a necessity.

A clear reporting of quality control methods in developmental neuroimaging studies is one important example of how to increase transparency. But given the high level of complexity of (developmental) neuroimaging studies, there are many other facets of a study that need to be reported in detail to properly evaluate and potentially replicate a study. The Committee on Best Practices in Data Analysis and Sharing (COBIDAS) within the Organization for Human Brain Mapping (OHBM) published an extensive list of items to report (see Nichols et al., 2017), and recently efforts have been made to make this a clickable checklist that automatically generates a method section (https://github.com/Remi-Gau/COBIDAS_chckls). We encourage both authors and reviewers to use guidelines such as COBIDAS or another transparency checklist (e.g., Aczel et al., 2019) in their work, in order to increase methodological transparency and the potential for replication of study results.

### 3.3 Addressing analytical flexibility through specification curve analysis

A potential solution to addressing flexibility in scientific analyses is to conduct a specification curve analysis (Simonsohn et al., 2015), a method that takes into account multiple ways in which variables can be defined, measured, and controlled for in a given analysis. The impetus for developing the specification curve analysis approach was to provide a means for researchers to present the results for all analyses with “reasonable specifications,” that are: informed by theory, statistically valid, and not redundant with other specifications included in the set of analyses run in a given specification curve analysis (Simonsohn et al., 2015). This approach presents a way for researchers with dissimilar views on the appropriate processing, variable definition or covariates to include in a given analysis to address how these decisions impact the answer to a given scientific question. This will give more insight in the *robustness* of a given finding, that is the consistency of the results for the same data under different analysis workflows (The Turing Way Community et al., 2019).

Specification curve analysis is an approach that works well for large datasets, and is gaining popularity within developmental science (e.g., Orben and Przybylski, 2019a, 2019b; Rohrer et al., 2017) and neuroimaging research (Cosme et al., 2020). To conduct a specification curve analysis, researchers must first decide on the reasonable specifications to include within a given set. Although specification curve analysis is often viewed as an exploratory method, the decisions regarding what to include within a given analysis can be preregistered (e.g., https://osf.io/wrh4x). Running a specification curve analysis does not mean that the researcher must include (or even could include) all possible ways of approaching a scientific question, but rather it allows the researcher to test a subset of justifiable analyses. The resulting specification curve aids in understanding how the variability in specifications can impact the likelihood of obtaining a certain result (i.e., can the null hypothesis be rejected). Each specification within a set is categorized as demonstrating the dominant sign of the overall set, which allows researchers to assess whether the variability in analytic approaches resulted in similar estimates for a given dataset. Running bootstrapped permutation tests that shuffle variables of interest can then be used to generate a distribution of specification curves when the null hypothesis is true. This can then be compared to the number of specifications that reject the null hypothesis in a given specification curve analysis.

To provide an application of specification curve analysis in developmental cognitive neuroscience one could for example address the multiplicity of ways how cortical thickness relates to well-being in young adolescents across the ABCD, Healthy Brain Network, and Philadelphia Neurodevelopmental Cohort. Cortical thickness can be estimated using several software packages, which can lead to considerably different regional thickness measures (Kennedy et al., 2019). If, for instance, the estimates generated by CIVET 2.1.0, Freesurfer 6.0, and the ANTS cortical thickness pipeline are used, this creates 3 possible ways of assessing cortical thickness within the set of specifications included in this specification curve analysis. But also, adolescent well-being can be assessed with different questionnaires and scales (Orben and Przybylski, 2019b), and the number of specifications will be limited by the instruments included in a given study. Finally, the relevant covariates to include in the analysis need to be specified and addressed in the analysis.

### 3.4 Reaching transparency through preregistration and registered reports

An effective solution to decrease biases in data analysis that can lead to inflated results is to use preregistration, in which the research questions and analysis plan are specified before observing the research data (Nosek et al., 2018). In this way, several biases are avoided that can easily lead researchers to HARKing or to see results as predictable only after seeing the actual results (hindsight bias). Note that preregistration is only used for *confirmatory* research planned to test hypotheses and for which one has specific predictions. It does not preclude *exploratory* research used to generate new hypotheses. Instead preregistration clearly separates confirmatory from exploratory analyses, thereby increasing the credibility of research findings. In the case of neuroimaging, one can think of specifying the analysis pipelines and regions of interest in advance, thereby eliminating the possibility of trying out different strategies that lead to inflated significant results by chance. Extensive information on preregistration and how to start registering your own study can be found on the Center for Open Science website (https://cos.io/prereg/). This can be done using basic preregistration forms that answer brief questions about study design and hypotheses (e.g., http://aspredicted.org/). There are also more extensive preregistration templates available specifically aimed at preregistering fMRI studies (see Flannery, 2018; and see also Table 1, for more resources on preregistration and Registered Reports).

With the growing number of open datasets, and for efficient reuse of existing datasets, preregistration for secondary data analysis also becomes more common. With existing data it might be harder to prove that one has not tested some of the preregistered hypotheses before preregistration, but this argument can in principle also be used for preregistrations of primary data. Preregistrations are partly based on trust, and dishonest researchers can theoretically also find ways to preregister studies that have already been conducted (Weston et al., 2019). In addition, in ongoing studies like the ABCD study, analyses can be preregistered for upcoming data releases. A time-stamped preregistration does in that case show that the researcher has not looked at the data yet. But in general, preregistration does not provide watertight guarantees that a researcher has not looked at the data or that the quality of the research is necessarily high-class. However, a well-written and thought-out preregistration for the analysis of existing data increases transparency of the analyses and reduces the risk of counterproductive cherry picking and data-fishing expeditions.

Some of the problems with preregistration may be solved using a more extensive form of preregistration, namely a *Registered Report* in which the preregistration is submitted to a journal before data collection (Chambers, 2013; for more information see https://cos.io/rr/). Hence, Registered Reports can be thought of as preregistrations that are peer reviewed. Consequently, modifications and improvements of the study plan can be made prior to actual data collection. In addition, once the proposal is approved, the paper receives an ‘in principle acceptance’ before any data is collected, analyses are performed or results are reported. Therefore, publication bias is eliminated since publication is independent of the results of a given study. Despite the advantages of external feedback and in principle acceptance of the manuscript, one of the drawbacks of Registered Reports for researchers is that it can take more time to start data collection. On the other hand, time is saved after data collection as large parts of the manuscript are already prepared and reviewed, there is also no need to engage in time intensive ‘journal shopping’. As argued above, Registered Reports can be very useful to decrease analytical flexibility of confirmatory studies with smaller datasets and in large-scale (openly available) datasets. Although Registered Reports usually need to be submitted before data collection at a moment that researchers can still revise the study’s methods, there are also possibilities for Registered Reports in the context of existing datasets. For example, after the first data release of the ABCD study, the journal *Cortex* hosted a special issue on Registered Reports for this ongoing study (http://media.journals.elsevier.com/content/files/cortexabcd-27122755.pdf). Authors were asked to propose hypotheses and analysis plans for the upcoming data release, with the possibility to use the previous data release as a testing sample for exploratory hypothesis generation and pipeline validation. As of May 2020, the *Developmental Cognitive Neuroscience* journal also publishes Registered Reports, with the explicit opportunity to submit secondary Registered Reports after data collection but before data analysis (Pfeifer and Weston, 2020).

### 3.5 Interim summary

Flexibility in data collection, analysis and reporting is an important challenge to reproducibility and replicability, but increasing transparency in all stages of the research cycle could prevent or diminish much of the unwanted flexibility. Methodological challenges specific for developmental studies may, for instance, be mitigated with the use and detailed reporting of age-appropriate scan protocols (e.g., mock scanning, movie-viewing paradigms), and sophisticated longitudinal modeling. Apart from the methods being used, critical evaluation and possible replication of studies furthermore greatly benefit from accurate and transparent reporting of those methods. As an example, we discussed available opportunities to deal with participant motion and its consequences and how to report choices made in this analysis step. To formally and transparently test the impact of such different choices, an emerging analysis method with high potential within our field is specification curve analysis. Finally, preregistration can be used to increase transparency and minimize undesired flexibility both in single site and openly available (longitudinal) studies.

## 4 What can researchers at different stages of their careers do?

### 4.1 Early career researchers

There are many utilitarian, economic, cultural and democratic arguments to adapt reproducible and replicable research principles, but we likewise highlight personal gains as well as risks. Both the benefits and potential drawbacks of adopting new research practices have been previously discussed specifically for early career researchers (Allen and Mehler, 2019; Poldrack, 2019). As for the concerns, first, early career researchers typically have fewer financial opportunities and a greater pressure to quickly produce research results and publications. This may limit the possibility to collect sufficiently large amounts of data or the time to learn to use open science tools or practices. Second, early career researchers may lack access to already collected data, research assistants, or necessary computing facilities. Based on this, Poldrack (2019) suggests that early career researchers can pivot to research questions where they are able to make progress, focus on collaboration, use shared data, or focus on theory and methods. Practically, early career researchers could take advantage of sequential data peeking to reduce expenses in collecting data (see section 2.1), although assuming some risk that the data collected will be enough to generate a dataset of value to the researcher’s goals (e.g., pilot data that can at least establish feasibility for grant applications). The option to primarily turn to open datasets means that early career researchers cannot always work on the questions they might be most interested in, simply because the specific data needed has not been collected. For more methodologically oriented researchers this might not be a problem, but those who want to launch their career addressing specific, novel, hypotheses might be put at a disadvantage. As noted earlier, such researchers might opt for collecting new samples mainly suited for exploratory analysis, which should be clearly labeled as such and evaluated accordingly by reviewers and editors (see Flournoy et al., 2020, for how to distinguish more clearly between confirmatory and exploratory analysis in developmental cognitive neuroscience). Such exploratory studies may accommodate more exciting and complex designs, which can be followed up by larger well-funded, confirmatory studies.

### 4.2 Established researchers

Discussions related to traditional versus open science research practices often, albeit with many notable exceptions, tend to follow power structures in academia. It can be argued that established researchers, publishers, journals and scientific societies that have been successful in the current system have less incentive to change, or even financial interests in keeping current practices. However, in order to make large changes in our research practices and improve replicability and reproducibility of developmental cognitive neuroscience, grant agencies and research institutes, boards at universities, senior researchers and faculty play critical roles. Changes such as obligations for data sharing and shifting incentive structures have to be made - and are being made in many places - at policy and institutional levels. Implementation of reproducible research practices is now for a large part done by graduate students and others early in their career. Here, established researchers can make a difference by supporting open science principles for their students and for future research. For senior researchers this might be more important and feasible than, for example, to make all their past research open access post-hoc.

### 4.3 Stageless

There are also many reasons why reproducible science practices can be beneficial to individual researchers at all stages. In the long run, working reproducibly helps to save time, mistakes are easier to spot and correct early on, and diving back into a project after months or years is much easier (see Markowetz, 2015, for more ‘selfish’ reasons to work reproducibly). Despite the opportunities that these practices provide for researchers, many solutions require extensive training (e.g., learning new tools) and changes in existing workflows. Therefore, to make progress as a field towards increased reproducibility and replicability, every incremental step taken by an individual researcher is welcome. There are many possibilities for stepwise contribution towards the goal of increasing the quality of research; working reproducibly is not a matter of all-or-nothing.

Regardless of career stage, several practical tools and strategies can be implemented in order to increase reproducibility and replicability of our work. As reviewed by Nuijten (2019), in addition to adopting preregistration, conducting multi-lab collaborations, and sharing data and code, which we have discussed above, this also includes working to improve our statistical inferences. She argues that many of the current problems in psychological science might relate to the (mis-)use and reporting of statistics. Generally, the solution to this is to improve statistical training at all levels, which can be addressed through asynchronous access to openly available courses and workshop materials, but also through immersive training experiences such as workshops and hackathons (e.g., the Brainhack format; Craddock et al., 2016). There have been specific workshops and hackathons specific to working with data in developmental cognitive neuroscience, including several specifically focused on using the ABCD dataset. Further, there are often pre-conference workshops associated with Flux Congress focused on topics specific to developmental cognitive neuroscience such as longitudinal modeling and analyzing complex neuroimaging data across multiple age periods.

Open science can be promoted in nearly all our academic activities. First, faculty and senior researchers are encouraged to lead by example, taking steps to improve the reproducibility of the research their groups conduct. Second, faculty and lecturers can cover and discuss the replication crisis and open science practices in their teaching (from undergraduate to postgraduate level courses; see Parsons et al. (2019a)for an open science teaching initiative). Third, it is critical that supervision and mentoring foster accurate and complete reporting of methods and results and interpretations that account for shortcomings of the work. An increased focus on research questions, hypotheses and rigorous methods rather than on results would beneficially impact the commonly mentioned replicability and replication crisis. Fourth, established researchers, promotion and hiring committees and review boards can work towards changing the incentives system to promote reproducible practices. One concrete example is to include use and promotion of open science research practices as a qualification when announcing positions, and to use this as one of several criteria when ranking applicants, or when evaluating faculty for promotions. Similarly, research funders can include practices like data and/or code sharing, open access publication, replication, and preregistration as formal qualifications, and encourage or demand such practices upon funding research projects. Research funders can also tailor specific calls promoting open science. For example, the Dutch Research Council (NWO) has specific calls for replication studies. Finally, journals and research societies can implement awards for reproducible research or replications (e.g., OHBM Replication Award). Several of these suggestions have been included in the DORA declaration (https://sfdora.org/).

## 5 Conclusion

There are currently unprecedented possibilities for making progress in the study of the developing human brain. These opportunities to increase the reproducibility and robustness of developmental cognitive neuroscience studies are partly thanks to technological advances such as web-based technologies for sharing data and analysis tools (Keshavan and Poline, 2019). We realize that there are still many steps to be taken to realize the full potential of these advances, not in the least by slowing down the pace of the current system and changing incentives (Frith, 2020). But in the meantime, we can embrace many of the opportunities offered by the current “credibility revolution” in science (Vazire, 2018), some of which were discussed in the current paper. We would therefore like to end with the words of Nuijten (2019): “Even if you pick only one of the solutions above for one single research project, science will already be more solid than it was yesterday” (Nuijten, 2019, p. 538).

## 6 Citation diversity statement

Recent work in several fields of science has identified a bias in citation practices such that papers from women and other minorities are under-cited relative to the number of such papers in the field (Caplar et al., 2017; Dion et al., 2018; Dworkin et al., 2020; Maliniak et al., 2013; Mitchell et al., 2013). Here we obtained predicted gender of the first and last author of each reference by using databases that store the probability of a name being carried by a woman (Dworkin et al., 2020; Zhou et al., 2020). By this measure (and excluding self-citations to the first and last authors of our current paper), our references contain 18.8 % woman(first)/woman(last), 8.1 % man/woman, 17.4 % woman/man, 53.7 % man/man, and 2 % unknown categorization. We look forward to future work that could help us to better understand how to support equitable practices in science.

## Data statement

NA

## Declaration of Competing Interest

None.
